# A top-down approach for fabricating free-standing bio-carbon supercapacitor electrodes with a hierarchical structure

**DOI:** 10.1038/srep14155

**Published:** 2015-09-23

**Authors:** Yingzhi Li, Qinghua Zhang, Junxian Zhang, Lei Jin, Xin Zhao, Ting Xu

**Affiliations:** 1 State Key Laboratory for Modification of Chemical Fibers and Polymer Materials, College of Materials Science and Engineering, Donghua University, Shanghai, 201620, People’s Republic of China

## Abstract

Biomass has delicate hierarchical structures, which inspired us to develop a cost-effective route to prepare electrode materials with rational nanostructures for use in high-performance storage devices. Here, we demonstrate a novel top-down approach for fabricating bio-carbon materials with stable structures and excellent diffusion pathways; this approach is based on carbonization with controlled chemical activation. The developed free-standing bio-carbon electrode exhibits a high specific capacitance of 204 F g^−1^ at 1 A g^−1^; good rate capability, as indicated by the residual initial capacitance of 85.5% at 10 A g^−1^; and a long cycle life. These performance characteristics are attributed to the outstanding hierarchical structures of the electrode material. Appropriate carbonization conditions enable the bio-carbon materials to inherit the inherent hierarchical texture of the original biomass, thereby facilitating effective channels for fast ion transfer. The macropores and mesopores that result from chemical activation significantly increase the specific surface area and also play the role of temporary ion-buffering reservoirs, further shortening the ionic diffusion distance.

Supercapacitors are a promising alternative energy technology; they can provide higher power densities, faster charge/discharge rates and longer lifespans because their energy storage mechanism involves reversible adsorption of electrolyte ions onto activated materials for electrostatic charge storage^1^. Charge separation occurs because of polarization at the electrode–electrolyte interface, producing what Helmholtz described in 1853 as the double-layer capacitance C: C =ε _r_ε_0_A/*d*, where ε_r_ is the electrolyte dielectric constant, ε_0_ is the dielectric constant of vacuum, *d* is the effective thickness of the double layer (i.e., the charge separation distance) and A is the electrode surface area^2^,^3^. A high specific surface area (SSA) facilitates high capacitance, which has spurred the development of nanoscale electrode materials^4^,^5^,^6^,^7^,^8^. However, the experimentally measured capacitance of nanoscale carbon electrodes has been far below its theoretical value because the accessible SSA is substantially smaller than the SSA of the pristine electrode material^9^,^10^. The insufficiently open porous structures influence the diffusion pathways of ions in the matrix, and the poor wettability adversely affects the adsorption of ions onto the surface of the electrodes, thereby limiting the utilizable SSA^11^,^12^. Li *et al.*^13^ fabricated graphene films using a bio-inspired self-stacked approach and demonstrated that a solvated graphene film exhibited the highest capacitance and rate capability among the investigated graphene films. The solvation process used water molecules as an effective "spacer" to prevent the restacking of graphene nanosheets to retain the high accessible SSA and effective ion diffusion. Later work further confirmed that graphene films employ a nonvolatile liquid electrolyte as a "spacer" to improve the accessible SSA, leading to a significantly enhanced volumetric energy and power capability^14^. Furthermore, on the basis of the mechanism of electrostatic charge storage, double-layer supercapacitors should exhibit a high power density. In practice, however, many electrodes exhibit relatively low rate capabilities^15^. For example, a modified graphene film electrode was observed to retain half of the capacitance of the pristine electrode material when the current density was increased from 1 to 8 A g^−1^ 16. This behaviour arises from the limitation of charge transport in the electrode or of ionic transport in the electrolyte^17^. The complex diffusion pathways exacerbate the limitation of ion transport at a high scan rates, delaying the formation of double-layer capacitance^18^,^19^,^20^,^21^,^22^. Therefore, obtaining a good diffusion pathway is the critical issue that must be solved to enable the fabrication of supercapacitors with excellent electrochemical performance.

Unfortunately, the synthesis of functional nanostructures often involves expensive starting materials and elaborate processing, both of which present a challenge for successful implementation in low-cost applications. By contrast, biomass has the delicate hierarchical structures necessary for the diffusion of electrolytes in living organisms, inspiring researchers to develop cost-effective routes for using biomass to prepare nanostructured electrode materials. For instance, ordered channel arrays of hollow carbon nanofibres originating from crab shells were used to encapsulate silicon to form anodes for Li-ion batteries. This composite electrode exhibits high energy and power densities and good cycling stability because of the short diffusion pathway of ions and stable skeleton structures^23^. Chitin, chitosan, glucosamine, polysaccharide and protein from biomass have naturally porous structures that can be carbonized to obtain activated carbon with unique porous structures^24^,^25^,^26^,^27^,^28^,^29^. Electrodes fabricated using these bio-carbon materials exhibit good rate capability and stable cycleability^30^,^31^. However, high-temperature carbonization leads to a reduction in the biomass and the partial destruction of pristine microstructure, decreasing the specific capacitance^28^. Chemical activation is a typical method for modifying carbon materials to improve their porosity^17^. For instance, activating agents such as NaOH, KOH and Na_2_CO_3_ have been used to etch carbon materials, resulting in the fabrication of a series of micropores, mesopores and macropores that optimize the structure and improve its application performance^32^,^33^.

In this work, we chose sisal leaves as a precursor to bio-carbon because of their strong mechanical properties, which facilitate the preparation of free-standing carbon monoliths. The resultant bio-carbon materials with a hierarchical texture inherited from sisal leaves were prepared under various carbonization conditions, which structures afford effective diffusion pathways for ions. Meanwhile, controlled chemical activation was used to manipulate the macropores and mesopores on the skeletons of the bio-carbon. Macroporous and mesoporous cores can be used as ion-buffering reservoirs to significantly improve the SSA and further shorten the diffusion distances to the interior surfaces. The free-standing electrode exhibited a high specific capacitance of 204.1 F g^−1^ at 1 A g^−1^; a good rate capability, including a residual initial capacitance of 85.5% at 10 A g^−1^; and a long cycle life. These results were likely due to the larger accessible SSA and the shorter diffusion pathways that promote both the adsorption of a greater number of ions onto the electrode/electrolyte interface and faster ionic transport.

## Results and Discussion

### Morphology and structure

**Figure 1:**
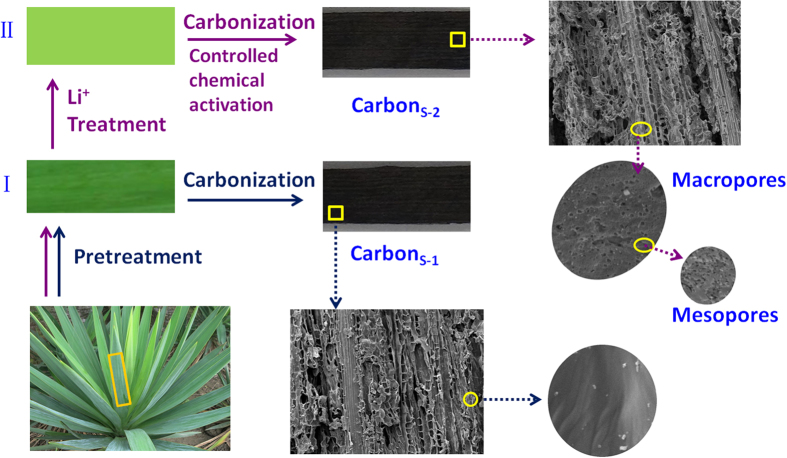
Schematic of the Carbon_S_ fabrication. Method (I) is direct carbonization to obtain the bio-carbon material monolith (Carbon_S-1_) that inherits the natural hierarchical texture of sisal leaves. Method (II) is to manipulate the free-standing bio-carbon with a rich porous substructure by carbonization via controlled chemical activation (Carbon_S-2_).

**Figure 2:**
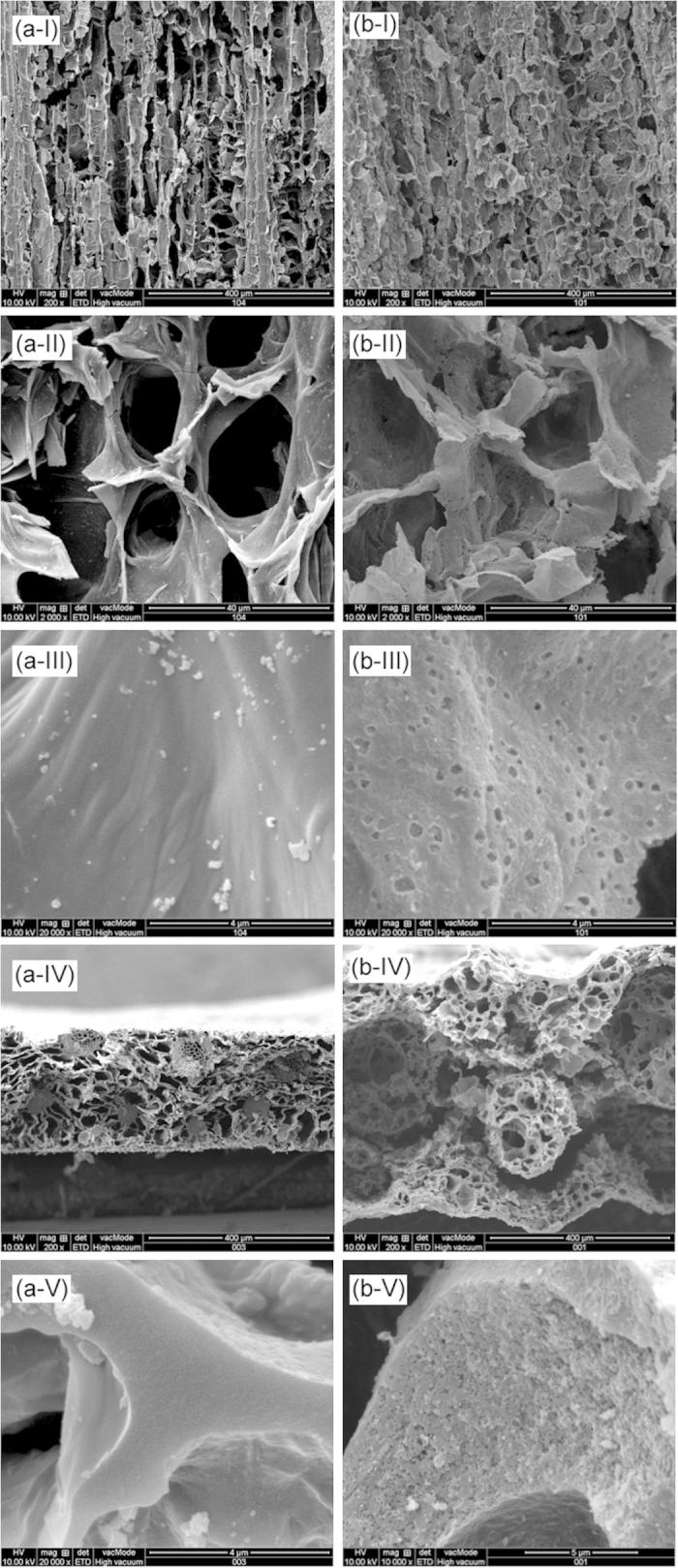
SEM images of the longitudinal surface (I, II, III) and the cross-section (IV, V) of Carbon_S-1_ (a-) and Carbon_S-2_ (b-). Carbon_S-1_ and Carbon_S-2_ inherited the pristine hierarchical porous frameworks of sisal leaves. Carbon_S-2_ was etched by controlled chemical activation to manipulate the macro/mesoporous substructures.

**Figure 3:**
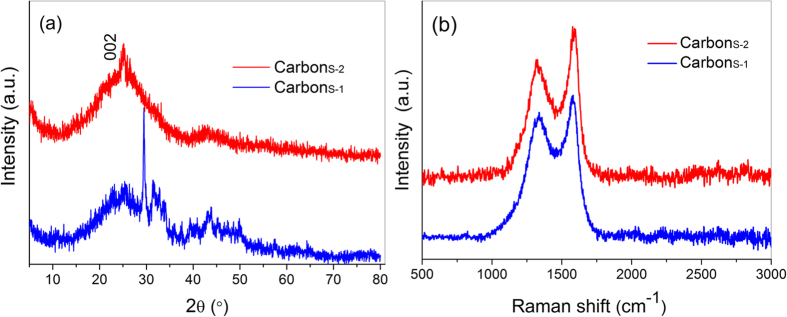
XRD patterns (a) and Raman spectra (b) of Carbon_S-1_ and Carbon_S-2_, which exhibit partial graphitic structures after carbonization.

Sisal hemp is a species of plant of the tropics and subtropics and consists of a rosette of sword-shaped leaves. Each sisal leaf contains an average of approximately 1000 fibres, which account for only approximately 4% of the plant by weight but help the leaves maintain their strong texture^34^. Because of its good mechanical properties and porous structure, sisal leaf is a promising candidate for the fabrication of free-standing carbon monoliths. Figure 1 displays a schematic of the processes for preparing bio-carbon from sisal leaves. One method is to directly prepare the bio-carbon material (Carbon_S-1_) by carbonization at high temperatures. Scanning electron microscopy (SEM) images of Carbon_S-1_ show connected porous frameworks with various pore sizes (Fig. 2a(I,II,III)) implying that Carbon_S-1_ inherited the pristine hierarchical structure of sisal leaves. SSA measurements confirmed that the Carbon_S-1_ BET surface area was as high as 171 m^2^ g^−1^ (Fig. 3a). Compared to the low BET values of the reported bio-carbons obtained without chemical activation^28^,^35^, our Carbon_S-1_ exhibits a relatively high surface area. This result is attributed to the novel freeze-dry pretreatment, which perfectly retains the organization of the sisal leaves’ structure after the removal of water from the biological body. To further improve the SSA and porosity, we used controlled chemical activation to fabricate microstructures on the bio-carbon. As shown in Fig. 1(II), the pretreated sisal leaf adsorbed ions from the solution containing lithium ions to serve as a precursor for use with activating agents. The pre-prepared sisal leaves were carbonized at high temperature to obtain the free-standing bio-carbon with a porous substructure (Carbon_S-2_). The types of activating agents and the activating agent/precursor ratio are known to be important factors for the manipulation of the porous structure during the chemical activation process^36^. Here, an optimized mixing solution of lithium hydroxide and lithium carbonate was used as an activating agent (see Supporting Information Figure S1 for the detailed procedure). The freeze-dried sisal leaves were immersed into the mixing solution to absorb the Li^+^, OH^−^ and CO_3_^2−^ ions. During the high-temperature treatment process, LiOH and Li_2_CO_3_ reacted with carbon to produce Li_2_O, H_2_O, CO, CO_2_, and organic molecules, and the framework of bio-carbon was etched to produce the rich porous microstructures.

The SEM images of Carbon_S-2_ presented in Fig. 2b(I,II,III) show that the connected porous frameworks with various pore sizes are similar to the morphology of Carbon_S-1_, demonstrating that Carbon_S-2_ also inherited the hierarchical structure of sisal leaves. Specifically, the introduction of moderate activating agents did not destroy the pristine skeleton of sisal leaves during the carbonization because of the controllable etching of the mixed activating agent of LiOH and Li_2_CO_3_. The surfaces of the Carbon_S-2_ pore walls differ from those of Carbon_S-1_, exhibiting a rich porous structure (Fig. 2b(III,V)). The thickness of Carbon_S-2_ was approximately 600 μm (Fig. 2b(IV)), which is considerably larger than the 150 μm thickness of Carbon_S-1_. Higher-magnification SEM cross-section images show the pore walls densely interspersed with pores at the meso/microscale (Fig. 2b(III,IV)). These macro/mesopores dramatically improve the SSA and also act as ion-buffering reservoirs that shorten the diffusion distance of ions during charge/discharge processes and promote a high rate capability. Although chemical activation significantly changes the morphology of bio-carbons, elemental analysis demonstrated that the Carbon_S-2_ components were similar to the Carbon_S-1_ components: the carbon content of Carbon_S-2_ (C: 77.8 wt%) was slightly lower than that of Carbon_S-1_ (C: 80.1 wt%, Table S1).

The XRD patterns in Fig. 3(a) show a pronounced broad (002) peak at 2θ = 25.4°, indicating that Carbon_S-1_ and Carbon_S-2_ both possess good graphitic structure even after chemical activation^37^. The interlayer spacing, *d*_002_, was calculated to be 0.350 nm using the Bragg equation; this interlayer spacing is slightly greater than that of natural graphite (0.335 nm) because of heteroatom doping in the graphite sheet^38^. The sharp diffraction peak at 2θ = 29.7° in the XRD pattern of Carbon_S-1_ is attributed to the salt derived from biological electrolytes in sisal leaves. This phenomenon is consistent with the higher-magnification SEM images (Fig. 2a(III,IV)), which display some salt particles. By contrast, no sharp peaks appear in the XRD pattern of Carbon_S-2_, implying that the residual lithium compounds originating from activating agents during carbonization were completely removed in the water-washing process.

Raman spectroscopy is a very versatile tool for characterizing carbon materials. It is sensitive to key materials properties such as doping and defect density. As shown in Fig. 3(b), two typical peaks were observed in the Raman spectra at approximately 1338 and 1589 cm^−1^; these peaks are attributed to the well-defined D-band and G-band, respectively^39^. The D-band arises from the doubly resonant disorder, and its intensity is strongly associated with the degree of disorder in the graphitic plane^40^. For the bio-carbons derived from sisal leaves, oxygen-rich groups in the biomass give rise to defects and the other phenomena such as nitrogen doping during carbonization, giving rise the disordered configurations. The G-band is related to the in-plane vibration of sp^2^ carbon atoms, which is a doubly degenerate (TO and LO) phonon mode (E_2g_ symmetry) at the Brillouin zone centre^41^. The sharp G-bands lead to an intensity ratio between the G-band and D-band (I_G_/I_D_) of 1.17 and 1.30 for Carbon_S-1_ and Carbon_S-2_, respectively, demonstrating the relatively high graphitization of these materials. Therefore, the moderate chemical activation did not destroy the graphitic structure of Carbon_S-2_.

**Figure 4:**
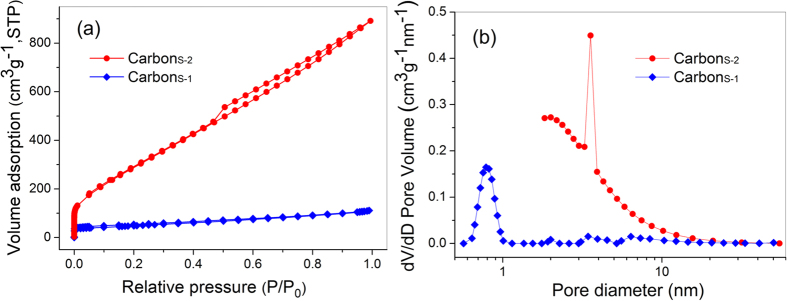
Nitrogen adsorption–desorption isotherm (a) and pore-size distribution (b) of Carbon_S_. The BET surface areas of Carbon_S-1_ and Carbon_S-2_ are 171 and 1173 m^2^ g^−1^, respectively. The total pore volume and predominant pore size of Carbon_S-2_ are 1.37 cm^3^ g^−1^ and 4 nm, respectively.

The nitrogen adsorption/desorption isotherms reveal differences in the pore structures and SSAs of the two samples (Fig. 4). Carbon_S-1_ exhibits a type-I isotherm with a H4 hysteresis loop, indicating a microporous structure^42^. On the basis of the Brunauer−Emmett−Teller (BET) equation, its SSA was estimated to be 171 m^2^ g^−1^, which is much higher than the SSA of some carbon materials not subjected to hydrothermal or chemical activated process^35^,^43^. The pore size calculated from adsorption data using the nonlocal density functional theory (DFT) method revealed that the Carbon_PH-1_ pore size distribution was centred at 0.8 nm, with a range of approximately 0.5–2.0 nm and a pore volume of 0.155 cm^3^ g^−1^. These results confirm the aforementioned SEM analysis results that indicated smooth pore walls because the pore size distribution closed the micropores that were not observed at current magnifications. After controlled chemical activation, the Carbon_S-2_ isotherm exhibited type-IV characteristics with a H3 hysteresis loop, which is associated with the occurrence of capillary condensation (Fig. 4a), indicating the presence of mesopores^42^,^44^. The BET surface area and the Langmuir surface area for Carbon_S-2_ were 1173 and 1985 m^2^ g^−1^, respectively. The total pore volume was 1.37 cm^3^ g^−1^. Moreover, the pore size distribution was narrow (Fig. 4b), with the predominant and average pore sizes calculated from the adsorption branch being ∼4.0 and ∼3.8 nm, respectively. The mesopore structure closing the micropores results in both shorter diffusion pathways of ion transport and an excellent BET surface area, improving the accessible SSA^33^,^45^,^46^. The higher accessible SSA and shorter diffusion pathways contribute to the high specific capacitance and high rate capability of the electrodes in supercapacitors.

### Electrochemical performance

**Figure 5:**
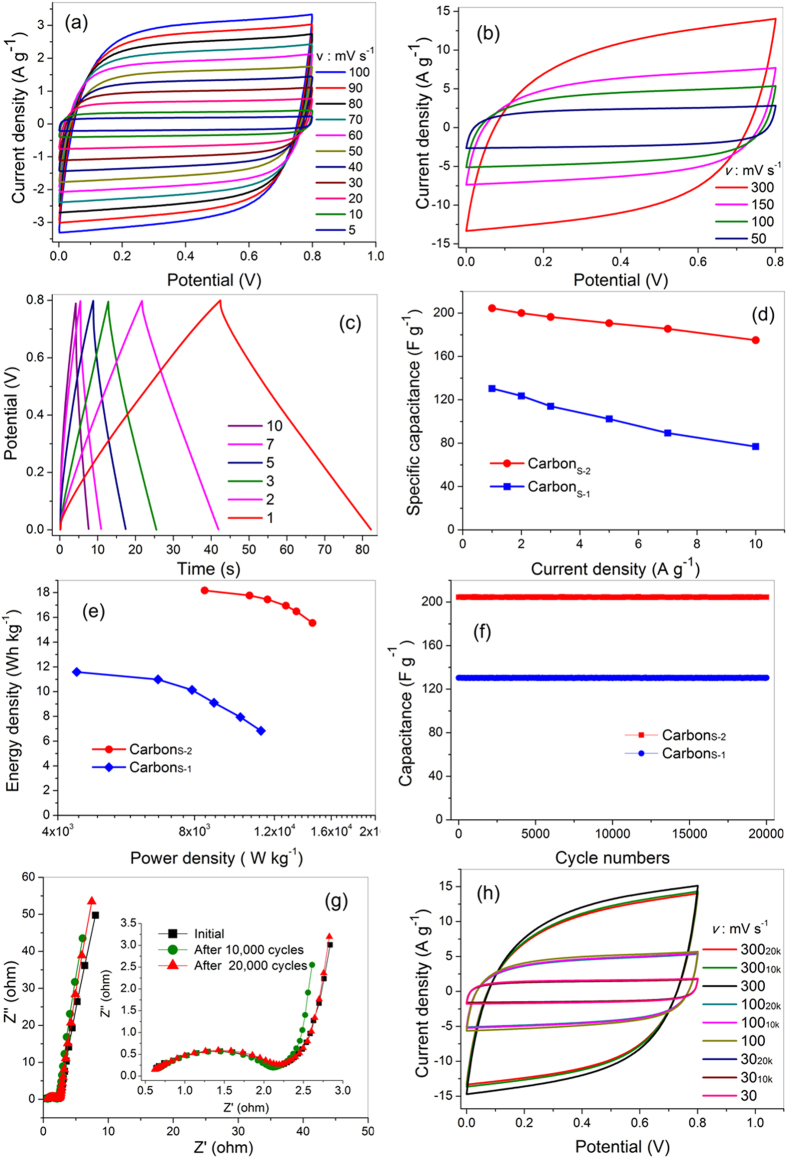
Electrochemical performance of Carbon_S-1_ and Carbon_S-2_: Cyclic voltammograms (a,b) of Carbon_S-1_ and Carbon_S-2_; galvanostatic charge/discharge curves (c) of Carbon_S-2_; rate capability (d), Ragone plots (e) and cycle life (d) of Carbon_S-1_ and Carbon_S-2_; Nyquist plots (g) and cyclic voltammograms (h) of Carbon_S-2_ at the first, 10,000^th^ and 20,000^th^ cycles.

The electrochemical performances of Carbon_S-1_ and Carbon_S-2_ were analysed in a symmetric two-electrode configuration. The cyclic voltammetry (CV) curves of the two samples exhibited perfect rectangle-like shapes, indicating a capacitive response derived from the EDL capacitance (Fig. 5a,b). The specific capacitance derived from the CV curves of Carbon_S-1_ slowly decreased with increasing scan rate (Figure S2b), demonstrating that its rate capability could be reasonably attributed to the facilitation of ionic diffusion by the hierarchical structures. Furthermore, Carbon_S-1_ exhibits stable capacitance characteristics. The capacitance and impedance are almost unchanged even for a three-fold increase in mass (Figure S2 c, d), making Carbon_S-1_ suitable for the fabrication of large-sized electrodes. Compared with the areas under the curves in the cyclic voltammogram of Carbon_S-1_, those in the cyclic voltammogram of Carbon_SH-2_ are clearly larger because of the improvement of the SSA by chemical activation and the resultant enhancement of the capacitance. Moreover, even at a fast scan rate of 300 mV s^−1^, the CV curve retained its rectangular shape, indicating the good power capability of Carbon_S-2_.

The galvanostatic charge/discharge curves exhibited a symmetrical triangular shape with a small IR drop (Fig. 5c), confirming the EDL capacitance. The IR drop was very small and did not obviously change with increasing scan rates, indicating the relatively low internal resistance and high Coulomb efficiency of the supercapacitor. These phenomena are closely related to the electrical conductivity of the electrode^47^. The conductivity of Carbon_S-2_, as determined using the four-point probe method, was 10 S cm^−1^, thereby verifying the good conductivity of this material. The galvanostatic charge/discharge curves also demonstrated the enhanced power capability of Carbon_S-2_. The capacitance slowly decreased from 204.1 to 175.0 F g^−1^ as the rate was increased from 1 to 10 A g^−1^. Thus, the retention ratio was 85%, and the residual capacitance of Carbon_S-1_ was 58 wt% (Fig. 5d). These results are attributed to the inherent hierarchical texture of bio-carbon inherited from sisal leaves and to the microstructures that originated from chemical activation. The hierarchal texture provides effective channels for fast ion transfer, facilitating the good rate capability. The chemical manipulation enabled modification of the multiscale pores, leading to a larger accessible SSA and to shorter diffusion pathways; these effects promote the adsorption of additional ions at the electrode–electrolyte interface and faster mass transport. The Ragone plots in Fig. 5e show that the modified hierarchical structure of Carbon_S-2_ gives rise to the higher specific energy density and power density observed experimentally. At 10 A g^−1^, the energy density of Carbon_S-2_ was more than two times that of Carbon_S-1_ and was accompanied by a greater power capability.

The Carbon_S_ electrodes exhibited outstanding cycling stability as double-layer capacitors (Fig. 5f), similar to the performance of other reported carbon materials^48^,^49^. The bio-carbons investigated in this work generally contain some hetero atoms such as nitrogen and oxygen (Table S1), promoting their wettability. However, on the basis of the aforementioned CV and galvanostatic charge/discharge curves, the nitrogen or oxygen functional groups attached on the bio-carbons appeared to barely affect the Faradic reactions during the charge/discharge processes. Thus, their pseudocapacitance can be ignored. Because the EDL storage mechanism does not involve the ions’ insertion/deinsertion, the devices theoretically have lifetimes that exceed one million cycles. However, the physicochemical properties and intrinsic structure of the electrode materials profoundly affect their cycling stability. Fortunately, the developed bio-carbons exhibit a stable intrinsic texture and physicochemical properties. After 20,000 cycles, the capacitances of Carbon_S-1_ and Carbon_S-2_ decreased on slightly. The SEM images collected after 10,000 and 20,000 cycles revealed that the original hierarchical structure (Figure S3) of the Carbon_S-2_ electrode was completely preserved. High-magnification SEM images revealed the presence of a homogeneous distribution of electrolyte salt particles on the Carbon_S-2_ surface. These phenomena demonstrate that the electrolyte can effectively diffuse onto the pore walls during the charge/discharge processes.

Electrochemical impedance spectroscopy (EIS) was used to investigate the change in resistance during the different galvanostatic charge/discharge cycling procedures. The equivalent series resistances (R_S_) and the charge transfer resistances (R_CT_) values of Carbon_S-2_ determined from the EIS measurements were smaller than those of Carbon_S-1_ (Fig. 5g, S2d), demonstrating that the chemical activation further improved the wettability to reduce the contact resistance and added mesoporous features to optimize the ionic diffusion pathways. Electrode materials with small R_S_ and R_CT_ values tend to enhance the power density of devices; thus, Carbon_S-2_ exhibited greater power capability than Carbon_S-1_ (Fig. 5e).

The 45° sloped portion of the Nyquist plots, referred to as the Warburg resistance, is a result of the frequency dependence of ion transport in the electrolyte^50^,^51^. The inherited hierarchical structure of the bio-carbons offers effective ion diffusion pathways; thus, the bio-carbons exhibited small Warburg resistances. In the low-frequency region, the Nyquist plots of Carbon_S_ show nearly vertical lines, indicating a pure capacitive behaviour^52^. The magnitude and slope of the impedance are associated with ionic diffusion in porous electrodes^19^. Carbon_S-2_, with its rich mesoporous structure, exhibited a smaller magnitude of impedance and more ideal capacitor behaviour. Moreover, the R_S_ and R_CT_ of Carbon_S-2_ barely changed with increasing number of charge/discharge cycles, confirming that the substructure and physicochemical properties were stable and did not deteriorate during the charge/discharge processes. The CV curves at 30, 100 and 300 mV s^−1^ after various numbers of cycles exhibit almost the same shapes and areas, further confirming the high stability of the electrochemical performance of Carbon_S-2_.

## Conclusions

We introduced the top-down approach for the fabrication of free-standing bio-carbon derived from sisal leaves by carbonization with controlled chemical activation. The bio-carbon inherited the pristine hierarchical texture of the sisal leaves and exhibited a rich porous substructure; the bio-carbon therefore exhibited a high SSA and a narrow pore size distribution. Because of its relatively high graphitization, this bio-carbon material also exhibited a high conductivity. As electrodes for supercapacitors, the free-standing bio-carbon exhibited a high specific capacitance of 204.1 F g^−1^ at 1 A g^−1^; good rate capability, as indicated by a residual initial capacitance of 85.5% at 10 A g^−1^; and a long cycle life. These results are attributed to the larger accessible SSA and to the effective diffusion pathways derived from the biomass, which promote greater ionic adsorption at the electrode–electrolyte interface and faster ionic transport, respectively. Therefore, our top-down approach using freeze-drying pre-preparation and controlled chemical activation offers an innovative method for the fabrication of bio-carbons that can be used as high-performance electrodes for supercapacitors.

## Experimental

### Synthesis of Carbon_S_

The cuticular wax of sisal leaves was removed and subsequently freeze-dried to preserve its pristine structure. The leaves were fixed by carbon fibre onto a plate of aluminium oxide and carbonized at 1000 °C in a tube furnace at a heating rate of 3 °C/min under a high-purity argon atmosphere. The precursors were annealed for 30 min at 1000 °C under the same atmosphere to obtain the bio-carbon materials (denoted Carbon_S-1_).

After the cuticular wax was removed and the sisal leaves were freeze-dried, the pretreated sisal leaves adsorbed ions from a mixed saturated solution of lithium hydroxide and lithium carbonate and were dried again. The pre-prepared leaves were carbonized under the aforementioned carbonization conditions to obtain the modified bio-carbon materials containing lithium compounds. For complete removal of residual lithium compounds, the sample was immersed in deionized water at 90 °C under high pressure for 24 h and was then washed until the wash solution was neutral (denoted as Carbon_S-2_).

### Materials characterization and electrochemical measurements

The morphology and structure of Carbon_S-1_ and Carbon_S-2_ were characterized by field-emission scanning electron microscopy (FESEM, Hitachi S-4800 and Quanta-250). The crystalline structure of Carbon_S-1_ and Carbon_S-2_ was characterized by X-Ray diffraction (XRD) measurements on a Rigaku D-max-2500 diffractometer using nickel-filtered Cu-Kα radiation with λ = 1.5406 Å. Fourier-transform infrared (FTIR) spectra were recorded on a Nicolet 8700 FTIR spectrometer. The spectra in the 4000–400 cm^−1^ range were collected by averaging of 32 scans at a resolution of 4 cm^−1^. Raman spectra were recorded using a LabRam-1B Raman spectroscope with He−Ne laser excitation at 632.8 nm; the scan time was 50 s. The BET surface area, pore volume and pore width of the bio-carbons were characterized by nitrogen adsorption using a surface area and porosity analyser (Quantachrome Autosorb-iQ). The specific surface area (SSA) was analysed using the BET method in the P/P_0_ range from 0.03 to 0.3; the pore volume was determined from the adsorption amount at P/P_0_ = 6.3 × 10^−7^ − 9.5 × 10^−1^, and the mesoporous volume and the mesoporous radius were obtained by the Barrett−Joyner−Halenda (BJH) method. The conductivities of Carbon_S-1_ and Carbon_S-2_ at room temperature were measured using a typical four-probe method (LORESTA-EP MCP-T360).

Three-electrode and two-electrode cell configurations were employed to measure the electrochemical performance. Carbon_S_ were first punched into wafer electrodes. Two nearly identical (by weight and size) electrodes were then assembled in a test cell. Electrochemical evaluations were performed on an auto-lab electrochemical workstation. The cyclic voltammograms and galvanostatic charge/discharge curves were collected from 0 to 0.8 V using 1 M LiOH as the electrolyte. EIS was performed for frequencies ranging between 100 kHz and 10 mHz using a perturbation amplitude of 5 mV versus the open-circuit potential. The electrochemical cycling tests of the products were performed using a LAND testing system; the electrodes were cycled more than 20,000 times.

## Additional Information

**How to cite this article**: Li, Y. *et al.* A top-down approach for fabricating free-standing bio-carbon supercapacitor electrodes with a hierarchical structure. *Sci. Rep.* **5**, 14155; doi: 10.1038/srep14155 (2015).

## Supplementary Material

Supporting Information
