# Supplementary material for: A top-down approach for fabricating free-standing bio-carbon supercapacitor electrodes with a hierarchical structure
Source: Sci Rep. 2015 Sep 23;5:14155. doi: 10.1038/srep14155 (PMC4585789; doi:10.1038/srep14155)
Supplement: Supporting Information [file srep14155-s1.pdf]

Supporting information

A top-down approach for fabricating free-standing bio-carbon supercapacitors electrodes with hierarchical structure

Yingzhi Li, Qinghua Zhang, Junxian Zhang, Lei Jin, Xin Zhao** **&** *Ting Xu*

State Key Laboratory for Modification of Chemical Fibers and Polymer Materials, College of Materials Science and Engineering, Donghua University, Shanghai, 201620, People's Republic of China. E-mail: qhzhang@dhu.edu.cn; Fax: 0086-21-67792854; Tel: 0086-21-67792854

Optimization of activating conditions

In order to improve the porosity of the bio-carbon, we carried out the chemical activation to modify the pristine structure. It is well know that the types of activating agents and the ratio of the activating agent/precursor are important parameters in chemical activation process.[1] In this work we did not adopt common activating agents such as KOH and NaOH, but LiOH and Li_2CO_3 to etch the bio-carbon. High-magnification SEM images demonstrate chemical activation does not destroy the pristine hierarchical texture and products the rich porous substructure (Figure S1c, e).

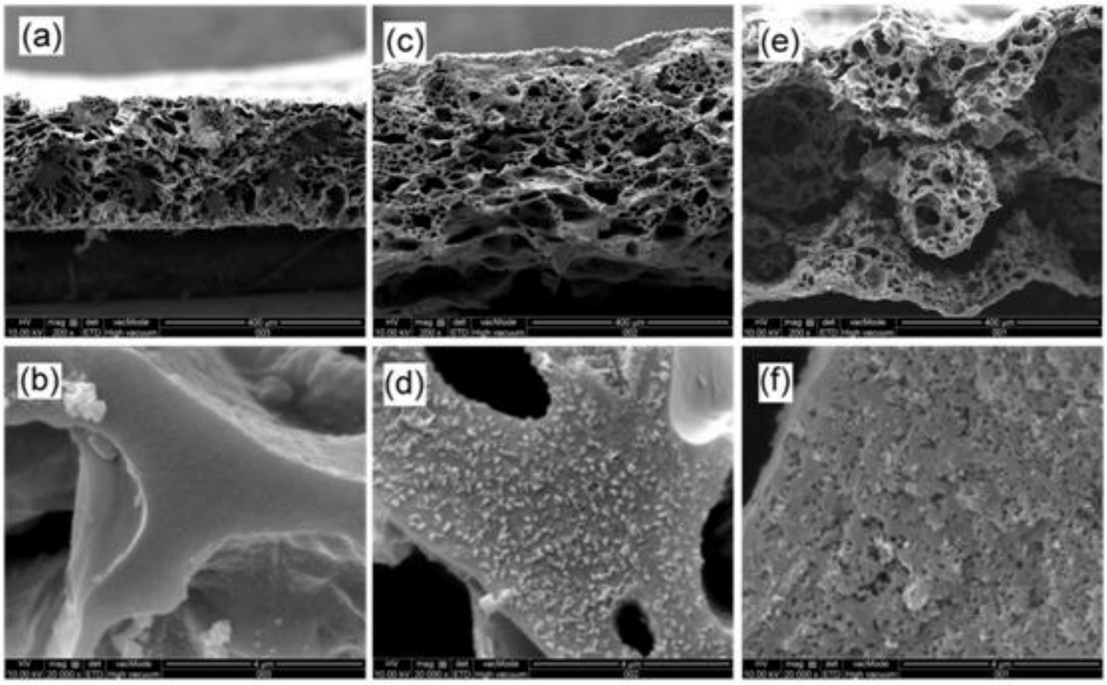

Figure S1. The cross-section images of $Carbon_{S-1}$ (a, b) with smooth pore walls. The cross-section images of chemical activated $Carbon_S$ (c, d derived from LiOH 1M; e, f derived from LiOH/Li_2CO_3 saturated solution) . After chemical activation, the thickness of bio-carbon dramatically increase and pore walls are etched to the rich substructures.

And the mixing solution exhibits the better etching effect than the single LiOH solution (Figure S1d, f). The thickness of bio-carbon dramatically increase from 150 to 600 μm when the precursor suffered from the rigorous etching effect of the saturated mixing solution of LiOH and Li_2CO_3 (Figure S1e). The results indicate the mixed solution of LiOH and Li_2CO_3 are expecting activating agents which can remain the inherent texture of bio-carbon and manipulates macro and meso pores on skeleton of the biocarbon as well, and control the predominant meso pores with small pore sizes.

Electrochemical performances of $Carbon_{S-1}$

The electrochemical performances of two bio-carbons were analyzed in a symmetric two-electrode configuration. Cyclic voltammetry (CV) tests show that all curves exhibit the perfect rectangle-like shape, indicating its capacitive response derived from the EDL capacitance (Figure S2a). The corresponding specific capacitance slowly decrease with the increase of the scan rate, demonstrating the rate capability is relatively good (Figure S2b). The connected porous frameworks inherited from biomass offer the effective diffusion pathways for electrolyte ions, facilitating the fast capacitive response of the electrode.

Generally, the specific capacitance decreases with the increase of the electrode mass.[2] In order to investigate the homogeneity of $Carbon_{S-1}$ structure, the $Carbon_{S-1b}$ electrode with three times mass of the $Carbon_{S-1}$ was tested the electrochemical performances. The CV curves reveal the specific capacitance of $Carbon_{S-1b}$ is 80.2 F g^{-1}, very close to that of $Carbon_{S-1}$ (Figure S2c). And Nyquist plots also exhibit the two electrodes with similar impedance spectra (Figure S2d). The results demonstrate the bio-carbon with uniform porous structure due to the stable and delicate structure controlled by genes of biomass. Meanwhile, The impedance spectra display the small equivalent series resistance (R_s from the X-intercept of the Nyquist plots), which comprises the resistance of the electrolyte solution, the intrinsic resistance of the active material, and the contact resistance at the interface active material/electrolyte.[3] It implies the good conductivity of the active materials. More importantly, the values of the charge-transfer resistance (R_{ct} from the diameter of semicircle of Nyquist plots at the real axis) are small, indicating the charge/discharge cycle processes are attributed to mass transfer control, not kinetic control.[4] Fabrication of the rich micro porous substructure shortens the diffusion pathways of ions, helping the decrease of $R_{ct.}$

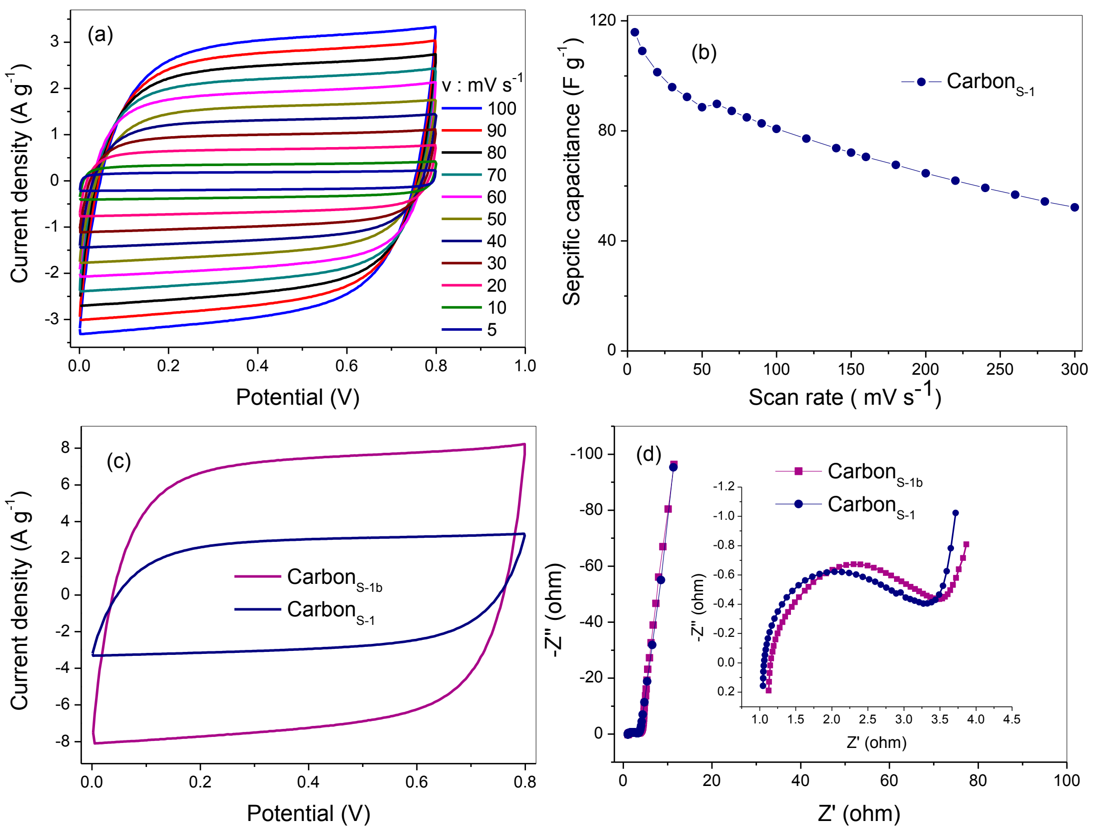

Figure S2. Electrochemical performances of $Carbon_{S-1}$. (a) Cyclic Voltammetry curves and (b) specific capacitances of $Carbon_{S-1}$. (c) Cyclic voltammetry at the scan rate of 100 mV s^{-1} and (d) Nyquist plots (from 100k to 0.01Hz) of electrodes of $Carbon_{S-1}$ and $Carbon_{S-1b}$.

Morphology of $Carbon_{S-2}$ after various cycles

As the Figure S3 shown, the electrode of $Carbon_{S-2}$ hold the hierarchical structure after 10,000 and 20,000 galvanostatic charge/discharge cycles, respectively. High-magnification SEM images further reveal the salt particles of LiOH electrolyte homogeneously distributing on the surface of $Carbon_{S-2}$, implying the electrolyte ions can effectively diffuse onto pore walls. The effective diffusion and the good wettability result in that $Carbon_{S-2}$ has the high specific capacitance and power capability.

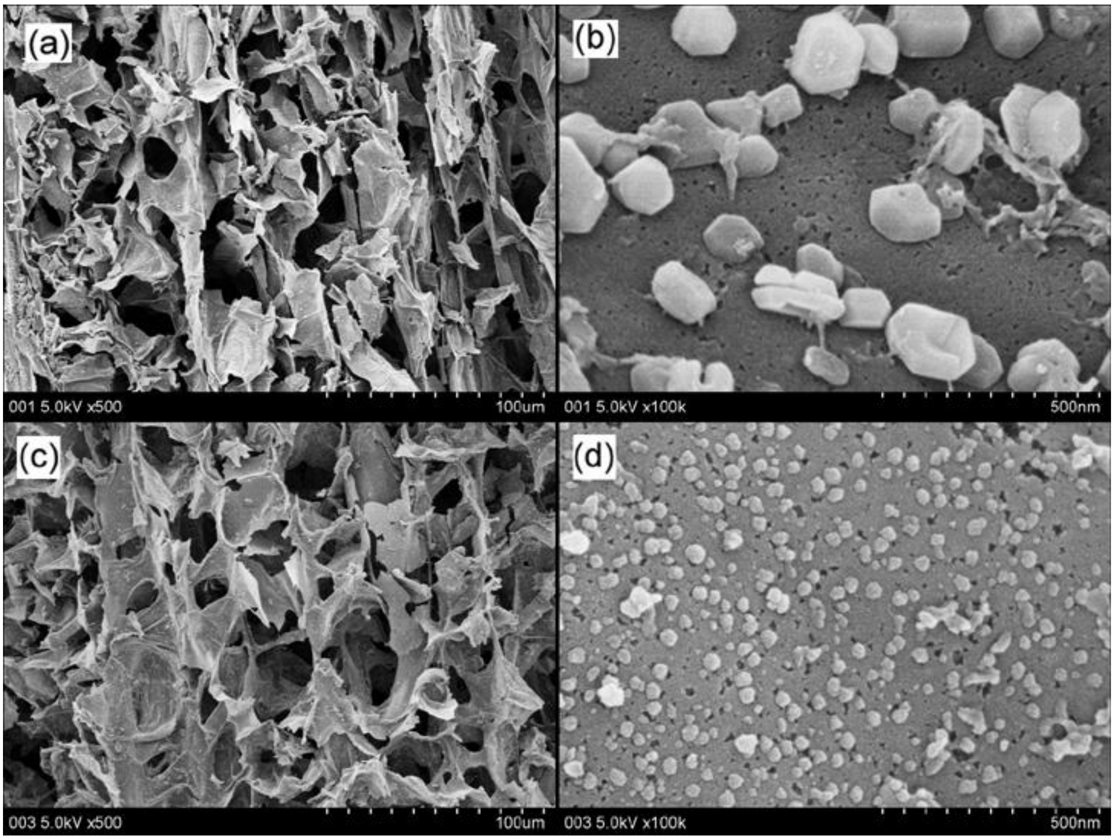

Figure S3. The longitudinal surface SEM images of Carbon$_{S-2}$ after 10,000 cycles (a, b) and after 20,000 cycles (c, d). High-magnification SEM images further reveal the salt particles of LiOH electrolyte homogeneously distributing on the surface of Carbon$_{S-2}$, implying the electrolyte ions can effectively diffuse onto pore walls.

Table S1 elemental analysis of Carbon$_{S-1}$ and Carbon$_{S-2}$

	C	H	N
Carbon$_{S-1}$	80.10%	1.53%	0.52%
Carbon$_{S-2}$	77.78%	1.35%	0.52%

Reference

1. Wang, H. *et al.* Nitrogen-doped porous carbon nanosheets as low-cost, high-performance anode material for sodium-ion batteries. *ChemSusChem* **6,** 56–60 (2013).

2. Zhao, X. *et al.* Incorporation of manganese dioxide within ultraporous activated graphene for high-performance electrochemical capacitors. *ACS nano* **6,** 5404–12 (2012).

3. Jiang, H., Zhao, T., Ma, J., Yan, C. & Li, C. Ultrafine manganese dioxide nanowire network for high-performance supercapacitors. *Chem. commun.* **47,** 1264–1266 (2011).

4. Bard, A. J., Faulkner, L. R., Swain, E. & Robey, C. *Fundamentals and applications*. Harris, D. New Yorke, USA (2001).
